# Barriers and enablers for adopting lifestyle behavior changes in adolescents with obesity: A multi-centre, qualitative study

**DOI:** 10.1371/journal.pone.0209219

**Published:** 2018-12-18

**Authors:** Maryam Kebbe, Arnaldo Perez, Annick Buchholz, Tara-Leigh F. McHugh, Shannon S. Scott, Caroline Richard, Charmaine Mohipp, Michele P. Dyson, Geoff D. C. Ball

**Affiliations:** 1 Department of Pediatrics, Faculty of Medicine & Dentistry, University of Alberta, Edmonton, Alberta, Canada; 2 Centre for Healthy Active Living, Children’s Hospital of Eastern Ontario, Ottawa, Ontario, Canada; 3 Faculty of Kinesiology, Sport, and Recreation, University of Alberta, Edmonton, Alberta, Canada; 4 Faculty of Nursing, University of Alberta, Edmonton, Alberta, Canada; 5 Department of Agricultural, Food, and Nutritional Science, Faculty of Agricultural, Life & Environmental Sciences, University of Alberta, Edmonton, Alberta, Canada; Leeds Beckett University, UNITED KINGDOM

## Abstract

**Background:**

Many adolescents with obesity do not meet recommendations for nutrition, physical and sedentary activities, and sleep habits, all of which can influence weight management.

**Objective:**

To explore barriers and enablers that influenced the adoption of lifestyle behavior changes among adolescents receiving multidisciplinary clinical care for pediatric weight management.

**Methods:**

In this multi-centre, qualitative description study, we used purposeful sampling to recruit 13–17 year olds (body mass index ≥85^th^ percentile) enrolled in one of two pediatric weight management clinics in Edmonton and Ottawa, Canada. Adolescents participated in one-on-one, in-person, semi-structured interviews in English or French. Interviews lasted 30–60 minutes, were audio-recorded, transcribed verbatim, and managed using NVivo 11. Data were triangulated using transcripts, field notes, and memos and analyzed by two independent researchers using inductive, semantic thematic analysis.

**Results:**

In total, 19 adolescents (12 Anglophone and 7 Francophone; 15.1±1.7 years old; 3.5±0.6 BMI z-score; n = 11 female; n = 13 Caucasian) participated. Adolescents reported diverse barriers to and enablers of healthy nutrition, physical and sedentary activities, and sleep habits, which we organized into the following themes: physiological mechanisms and physical health status, self-regulation for behavior change, controllability and competence beliefs, social relationships and interactions, and accessibility to and availability of opportunities for lifestyle enhancement. Across these themes and lifestyle areas, we identified three shared barriers and/or enablers, including the degree of controllability, the impact of mental health, and social pressures related to weight management.

**Conclusions:**

This research provides evidence that can be used to tailor interventions and health services delivery, including a focus on psychosocial well-being, to support adolescents with obesity in making and maintaining healthy lifestyle behavior changes.

## Introduction

Recent international data point to a ten-fold increase in the prevalence of pediatric obesity over the last four decades [1]. The complexity of obesity is particularly apparent during adolescence. Adolescents with obesity tend to experience a variety of psychological and social problems, including an increased risk for depression [2] and low self-esteem and quality of life [3] as well as difficulties in finding a partner [4] and delayed childbearing in adulthood [5]. Adolescent obesity is also concerning because it is associated with a higher risk of developing long-term adverse health consequences such as type 2 diabetes and cardiovascular disease [6] and is likely to be maintained in adulthood [7]. This extended exposure to obesity can have a negative impact on individuals as well as families, the health care system, and society.

Adolescents can derive health benefits from making healthy lifestyle and behavioral changes to prevent and manage obesity [8]. To help achieve these outcomes, adolescent-specific lifestyle recommendations have been developed, which include 6 to 8 daily servings of fruits and vegetables [9], 60 minutes of daily moderate-to-vigorous physical activity (MVPA) [10], no more than 2 hours of daily leisure-time sedentary activity [11], and 8 to 10 hours of sleep per night [11]. In Canada, a number of weight management programs exist [12] in which multidisciplinary teams deliver lifestyle and behavioral therapeutic interventions using a variety of behavioral change techniques (e.g., motivational interviewing [13], cognitive behavioral therapy [14]). There is growing evidence to support the effectiveness of lifestyle-based interventions for positive long-term changes in weight, especially those that combine behavioral, diet, and physical activity components [15]. Regardless of any weight change, adopting and maintaining a healthy lifestyle is important since it is an adjunct to other therapeutic options for the management of obesity (e.g., pharmacotherapy, bariatric surgery) and has been shown to lead to meaningful improvements in cardiometabolic risk factors, including low-density lipoprotein cholesterol, triglycerides, fasting insulin, and blood pressure [16]. Despite the advantages, many adolescents with obesity do not meet lifestyle behavior recommendations [17] and are at higher risk than their younger peers to drop out of weight management interventions [18], which may be a result of experiencing less success in weight management [19]. A guiding principle in pediatric weight management includes family centeredness [12], which acknowledges the consistent role played by parents in supporting adolescents’ health and wellbeing. However, adolescents’ perspectives may sometimes go unnoticed or be taken for granted [20]. To meet the needs of adolescents with obesity in weight management, it is imperative to gain a better understanding of their experiences in trying to change their lifestyle behaviors, especially with respect to the barriers and enablers that influence their ability to make changes.

In a recent review, we synthesized adolescents’ barriers to and enablers of lifestyle behavior changes to manage pediatric obesity [21]. We identified and mapped a range of barriers and enablers for nutrition and physical activity habits across the Social Ecological Model; however, evidence is still limited. For example, most studies included in this review (i) were limited to adolescents living in the United States, (ii) included perspectives of Anglophones exclusively, (iii) focused on nutrition and physical activity with little attention given to other habits known to influence weight and health, including sedentary activity and sleep, and (iv) seldom examined impeding and enabling factors of lifestyle changes within multidisciplinary clinical settings designed to help adolescents in weight management. To address these knowledge gaps, we designed our study to explore barriers to and enablers of adopting healthy lifestyle behavior changes. We sought to address the following question: Which barriers and enablers exist for healthy nutrition, physical and sedentary activities, sleep habits, and mental health among Anglophone and Francophone adolescents with obesity seeking multidisciplinary clinical care for weight management?

## Methods

### Study design

This multi-centre, qualitative study was conducted from July 2017 to January 2018. We adhered to principles of patient-oriented research, a continuum of research that engages patients as partners and focusses on patient-identified priorities, with an overarching goal of translating knowledge to the point of care to improve patient outcomes [22]. It was also guided by qualitative description, a method that draws on components of naturalistic inquiry, which is well-suited to behavioral research and does not require detailed interpretive interference; rather, it gathers direct, practical insights from participants to develop a description of a phenomena and stay close to the data and surface of participants’ words [23]. We conducted this study in Canada’s two official languages to gain a better representation of adolescent experiences from both Anglophone and Francophone populations. Since we believe language to be a social construction, we followed a constructivist paradigm [24], which views reality as being socially constructed and multifaceted (relativist ontology) and places the researcher in an active role for generating data dependent on their understanding and knowledge of the world (subjective epistemology). We operationalized barriers and enablers as factors that, according to adolescents, prevented or supported the implementation (defined as initial adoption) and maintenance (defined as continued adoption) of lifestyle behavior changes. Ethical and operational approvals were granted by human research ethics boards from study sites in Edmonton and Ottawa, namely the University of Alberta, Alberta Health Services, and the Children’s Hospital of Eastern Ontario.

### Preliminary step–patient engagement panel

Before initiating data collection, we convened a patient engagement panel (PEP; see S1 Appendix for contract) with five adolescents with obesity. Adolescents were purposefully sampled from a local pediatric weight management clinic (Pediatric Centre for Weight and Health [PCWH]; Stollery Children’s Hospital, Edmonton, AB) and invited to attend a ~2h group discussion. Led by MK, the PEP consisted of a semi-structured bidirectional conversation with adolescents, including hands-on activities. The purpose of the PEP was to explore adolescents’ experiences in weight management, generate ideas, and gather feedback on elements of our study, including the scope, interview guide, study material, and logistic factors. Based on adolescents’ feedback, we finalized several decisions in planning our study, including exploring adolescents’ mental health in relation to lifestyle behavior changes, adopting a more lay approach to our interview guide, providing gift cards with widespread use (i.e., Visa), and not placing a limit on interview length to allow adolescents to express themselves freely.

### Participants and recruitment

We completed main data collection for this study at two sites: the PCWH and the Centre for Healthy Active Living (CHAL; Children’s Hospital of Eastern Ontario, Ottawa, ON). Both of these clinics are located in urban areas in Canada, offer long-term, patient- and family-centered, and multicomponent behavioral, dietary, and physical activity clinical care to families of children and adolescents with obesity via multidisciplinary teams composed of pediatricians, dietitians, exercise specialists, nurses, psychologists, and social workers. We used purposeful sampling to recruit adolescents who were (i) 13–17 years of age with a body mass index (BMI) ≥85^th^ percentile and (ii) receiving weight management for ≥3 months at the PCWH or CHAL, which helped to ensure that they had spent some time reflecting on their lifestyle behaviors and working with health care providers (HCPs) on weight management. We excluded adolescents presenting with known developmental disabilities as their experiences in changing their lifestyle habits may have been impacted by their condition(s); to better represent this group of adolescents, there is value in conducting individual research to gain a comprehensive understanding of their perspectives. To help recruit our sample, we displayed recruitment posters at each of the clinic waiting rooms. In addition, administrative, clinical, and research staff identified eligible adolescents and their families from clinic databases, who were then approached by MK or CM in-person or by telephone to gauge interest. We offered interested adolescents a range of dates and times to choose from to participate in our study. All adolescents who completed the interview received a $25 gift card as a token of appreciation; we chose this monetary amount based on positive anecdotal experience from previous research conducted by our team. To ensure adolescents did not feel coerced to participate in our research, they were made aware of the possibility to withdraw from the study at any point without affecting the treatment they receive at the clinic or their compensation.

### Data collection

We invited adolescents to participate in individual, semi-structured interviews (30–60 minutes in length) in either English (PCWH) or French (CHAL). The interview guide (Table 1) was informed by current literature and refined with input of the PEP and from members of our research team. The first author (MK) conducted all interviews since she is fluent in English and French and formally trained in qualitative research; she first explained the aims of the study to adolescents, highlighted the right to not answer questions they did not feel comfortable with, and provided an opportunity for questions. Interviews were digitally-recorded, uploaded to an online and secure file sharing platform (LabKey) maintained by the Women and Children’s Health Research Institute (UAlberta), and transcribed verbatim by the Translation Agency of Alberta, a group that offers transcription services in both English and French. In addition to maintaining an audit trail to document study progress, MK prepared field notes and memos immediately after each interview to capture observations beyond those from audio-recordings and aid with theme generation and integration between categories. These data, along with interview transcripts, provided a comprehensive overview of the interview and assisted with triangulation, whereby data were collected from more than one source, coded by two independent researchers, and discussed internally with team members. We collected self-reported demographic and objectively measured anthropometric data of adolescents from medical records before or after the interviews. We also collected in-person self-reported demographic, sociodemographic, and anthropometric data from their parents (for descriptive purposes) before the interviews. We obtained written and informed written consent or assent from parents and adolescents prior to data collection.

**Table 1:** Interview guide exploring barriers to and enablers of adopting healthy lifestyle behavior changes by adolescents with obesity.

**Introduction**
1. Describe what a typical day looks like for you.
**Nutrition**
2. Tell me what you eat or drink on a typical day.
3. What comes to mind when I say ‘healthy and unhealthy foods’?
4. Are there things that make it easy for you to eat healthy?
5. What about things that make it hard for you to eat healthy?
**Physical Activity**
6. Tell me about any physical activities that you do throughout the day.
7. What comes to mind when I say ‘physical activity’?
8. Are there things that make it easy for you to be physically active?
9. What about things that make it hard for you to be physically active?
**Sedentary Activity**
10. Tell me about the things that you do during the day when you are not physically active.
11. What comes to mind when I say ‘inactivity’?
12. Are there things that make it easy for you to be inactive?
13. What about things that help you not be inactive?
**Sleep**
14. Tell me about your sleep habits.
15. What comes to mind when I say ‘healthy sleep’?
16. Are there things that make it easy for you to have better sleep?
17. What about things that make it hard for you to have good sleep?
**Mental Health**
18. Some teens have told me that they like to come to the clinic to learn how to address certain issues like anxiety, depression, or family conflicts. Does this apply to you at all?
19. Are there things that have helped you talk and learn about or address these issues?
20. What about things that have made it hard for you to talk about and address these issues?
**Summary**
21. Is there anything that you’d like to add to what we talked about?

### Data analysis

We collected and analyzed data on an ongoing basis, informing additional data collection such as the addition and removal of specific probing questions. Once transcribed, we de-identified and checked transcripts for accuracy. In our interviews, we provided adolescents with the opportunity to select their own pseudonyms; otherwise, we selected names that would resonate with them [25]. Pseudonyms over characteristics of sex and age-range were chosen to facilitate following individual narratives [26]. We imported and managed our data using NVivo 11 (QSR, Melbourne, Australia), which we analyzed using inductive, semantic thematic analysis [27]; that is, we identified themes within the explicit meanings of the data. MK and AP independently read and re-read the first five transcripts for familiarization with the data. Both authors independently developed a coding scheme for barriers and enablers, then applied a refined version to the entire data set with new codes developed iteratively when necessary. The coding tree was organized by topic (e.g., barriers, enablers), root codes (e.g., nutrition, physical activity), and code names (e.g., motivation for change). Codes referring to the same barriers and enablers were grouped into themes and exemplar quotes were chosen to illustrate the developed categories. MK and AP held regular meetings to review and compare codes, themes, and quotes, which were finalized through discussions and refinements with other research members (CR, GB). Any discrepancies were resolved by consensus.

### Methodological rigor

We used several strategies to ensure methodological rigor, including investigator responsiveness (e.g., ongoing analysis), methodological coherence (e.g., congruence between the research question and method), sampling adequacy (e.g., data saturation), and theoretical thinking (e.g., reconfirming ideas emerging from data in new data) [28]. MK also examined her own role as a researcher through an ongoing critical reflection, including personal reflexivity (identity, interests, and values), functional reflexivity (nature of the study), and disciplinary reflexivity (field of inquiry) [29], and how these characteristics may have shaped the research process and influenced data collection and analysis. For example, MK’s own professional orientation and personal interests in health research and life and nutritional sciences may have created certain biases that were brought forth when listening to adolescents’ accounts of changing their lifestyle habits. Further, while she empathized with adolescents and was able to diminish power dynamics and establish rapport due to unimportant age differences (insider perspective), MK is of regular weight (outsider perspective), which may have hindered her ability to fully understand the efforts required by those with excess weight to adopt healthy lifestyle behaviors. As she became more immersed in the interviews, however, she progressively gained a better understanding of adolescents’ experiences surrounding lifestyle change.

We undertook recommended processes for translation in cross-language studies [30–33]. First, MK acknowledged how her identity and linguistic stance may have affected study processes and analyses, so once she translated study documents, including consent/assent forms, recruitment scripts, and interview guides, another team member and Francophone health researcher (CR) reviewed and refined these materials. To avoid potential mistranslations for data analysis, MK (i) analyzed interview data in the source language, (ii) discussed (with CR) decisions made related to data analysis in the source language, and (iii) confirmed accuracy of back-translation of concepts, categories, and quotes into the source language (TAA); those involved in the translation processes (MK, CR) reached consensus by discussion. We translated data from French to English to ensure concepts and categories matched between PCWH and CHAL sites and because we planned to publish our results in an English-language journal.

## Results

A total of 19 adolescents participated in our study. Most adolescents were female, Anglophone, Caucasian, lived with severe obesity, and had parents who met criteria for overweight and obesity (Table 2). No appreciable differences in regards to interview data were observed across clinics, so we grouped the reported barriers and enablers across lifestyle areas in nutrition, physical and sedentary activities, and sleep; issues related to mental health cut across the other lifestyle areas and themes. Themes included physiological mechanisms and physical health status, self-regulation for behavior change, controllability and competence beliefs, social relationships and interactions, and accessibility to and availability of opportunities for lifestyle enhancement. Across these themes and lifestyle areas, we identified three common factors that had a positive or negative impact on making healthy lifestyle behavior changes, including the degree of controllability, the impact of mental health, and social pressures.

**Table 2:** Demographic, anthropometric, and sociodemographic characteristics of adolescents and their parents.

	Adolescents	Parents
	(n = 19)	(n = 19)
Age (y)	15.1±1.7	49.5±9.0
Sex (n; %)		
*Female*	11; 57.9	13; 68.4
*Male*	8; 42.1	6; 31.6
Ethnicity, (n; %)		
*Caucasian*	13; 68.4	13; 68.4
*Non-Caucasian*	6; 31.6	6; 31.6
Household Income (>$50,000/y CDN) (n; %)	-	13; 72.2^a^
Height (cm)	164.7±7.0	164.9±11.0
Weight (kg)	103.8±16.7	83.7±14.4
Weight Status (n; %)		
*Normal Weight*	-	1; 5.3
*Overweight*	-	10; 52.6
*Obesity*	4; 21.2	6; 31.6
*Severe Obesity*	15; 78.9	2; 10.5
Body Mass Index (BMI; kg/m^2^)	37.9±4.1	30.8±5.2
BMI percentile	99.9±0.001	-
BMI z-score	3.5±0.6	-

Data presented as mean ± standard deviation unless otherwise specified.

^a^n = 18; one parent chose ‘prefer not to say’.

### Nutrition

#### Self-regulation for behavior change

Adolescents described finding it difficult to change established behaviors. As one adolescent stated: “I know I wouldn’t make healthy food myself […] If I made my own food it would just be a bunch of junk food.”–Bill

Difficulties in healthy eating were especially present for those who reported having anxiety related to food or eating disorders (bulimia nervosa). For example, one adolescent described enjoying food in the moment, which was followed by vomiting either foods or drinks (both more and less healthy options) to avoid weight gain: “It's not that it's like, the having healthy food choices, like a hard thing for me. It's just the fact that food is a hard thing for me […] I just think it’s like bad to eat, I guess.”–Nixy

This can be contrasted with some adolescents whose upbringing focused on healthy eating, predisposing them to preferring the taste of healthier foods. One adolescent described the process of behavior regulation as follows: “If you grew up from a life of like, just eating junk food and not a lot of healthy food, it’s pretty hard to adjust, and you got to maybe like, you got to do stuff you don’t like, and you have to know you’re not going to like it, but you still have to do it.”–Bill

#### Controllability and competence beliefs

A common barrier to a healthy diet was a perceived lack of controllability over eating patterns and preferences (e.g., taste, portion control, mindless eating). As one adolescent stated: “It’s easier to make healthy food choices if you like the food choice. You shouldn’t like, force anything into your mouth just to be healthy.”–Bill

Adolescents shared numerous examples to suggest that intrinsic motivation was a common enabler of a healthy diet. Most adolescents shared that change had to come from within themselves, and reported a number of factors that enhanced their motivation, including adequate knowledge of health and nutrition, likeability of the food, setting realistic and gradual goals, and not viewing change as dieting. The following example illustrates this: “Not thinking of it as a diet because if I think of it as a diet and try to restrict myself, it’s like I want it so bad because you’re trying to convince yourself in a diet, but if you’re in a diet it just makes you want the thing more.”–Daniel

#### Social relationships and interactions

Social barriers to healthy eating included a lack of parental involvement in behavior change, low perceived parental confidence in adolescents’ ability to change eating habits, and negative parental judgments about adolescents’ eating behaviors. For example, some adolescents reported feeling pressured to eat unhealthy foods in response to their parents’ or peers’ practices and expectations. One adolescent shared her experience: “Um, I don’t like, my Mom is kind of always trying to do her own thing for healthy eating, so she kind of just switches from like different diets or like plans or whatever and then she like will go and like random baking sprees where she’ll bake like all of this unhealthy stuff that’s just like packed with sugar and then she won’t eat any of it. She just wants everyone else to eat it and you can’t not eat it because then she’ll get offended.” She continued: “My mom a lot, like it bothers me, like bugs me a lot about what I eat, like how much of it I’m eating, and so like, I already kind of like, struggle with those issues myself, like kind of like beating up on myself for it […] so that like makes it really difficult because then it kind of just makes me feel bad and then also creates spite, and so then I end up eating more anyways in order to like spite her.”–Courtney

Adolescents were also subject to direct or indirect peer pressure to conform to social expectations in relation to eating. For example, while some described feeling pressured to eat unhealthy foods in social outings as a means of conformity, others reported being bullied for attempting to eat healthy foods: “Um, things that are stopping me they’re like sometimes people like to make, uh, fun of me for trying to like eat healthy. They’re like what are you doing? You’re never going to actually be able to eat healthy. Just do it the way you’re doing it right now. And whenever I try to confront someone after they keep telling me to start eating healthy they tell me it’s nonsense and that they’re never do that to me. So that’s kind of, people are what is stopping me kind of.”–Dipti

Social enablers of healthy eating included interpersonal support from family members, peers, and HCPs. This support came in the form of encouragement, active participation (e.g., role modeling), availability (e.g., abundance of healthy foods, limited access to unhealthy foods), and accommodation (e.g., premade foods). One adolescent shared the value of home visits as an example in helping to navigate the aforementioned factors: “Um, we like the one thing that really, really helps is when the dietitian and the physical activity nurse who help you, when they come to home and because they do home visits as well, so that really helps to manage our time and like that way I don't skip my school and things like those.”–Zaid

#### Accessibility to and availability of opportunities for lifestyle enhancement

The affordability, availability, and convenience of unhealthy foods in different settings (e.g., home, school, restaurants) were highlighted as barriers to healthy eating. At the home, some adolescents described being challenged by the availability of unhealthy foods and the lack of availability of healthy foods. Unhealthy foods were dictated by family members’ preferences, which adolescents reported having no control over. This visibility of unhealthy foods, coupled with an insufficient abundance of healthy foods at the home, the increased cost of healthy foods, and the presence of unhealthy foods in their surroundings (e.g., school cafeteria, workplace, restaurants) further added to the challenge of healthy eating. This is demonstrated by the following quote: “So it’s harder to like go to the store, buy an apple, if we don’t have any, and pay for it than it is to just like, I’m just going to grab a bag of chips because they’re about the same price and they taste better or just as good.”–Daniel

In some households, parents of adolescents ensured that a system was set in place for healthy eating. Adolescents described valuing this approach, which included availability of healthy premade foods at the home and the unavailability of unhealthy foods.

### Physical and sedentary activity

#### Physiological mechanisms and physical health status

Adolescents described multiple reasons for limited physical activity. For instance, adolescents experienced physical discomfort due to a number of factors (e.g., knee pain, feeling tired / lack of sleep, side effects of medications taken for mental health). As quoted: “I think, because I’ve always been bigger, like even when I was younger, I was skinny, but I was well-built. Like, I have big… I’ve got wide shoulders, broad hips, and all that. So, I don’t know. I find running uncomfortable and I’m busty, so when I run, it’s uncomfortable.”–Eliza

#### Self-regulation for behavior change

Many adolescents attributed their low levels of physical activity to preferential factors (e.g., dislike of organized sports, especially running), and commented broadly on their behavioral regulation: “Now I realize it’s not really my family stopping me. It’s me. So even though it’s like a bit easier now that I can, I have some opportunities. It’s just kind of me stopping myself from going.”–Dipti

When not active, adolescents described a preference to sedentary activity, especially using digital technology, including social media. Adolescents also attributed their sedentary behaviors to their upbringing, lack of parental monitoring, feeling bored, and immediate gratification and enjoyment.

Some adolescents shared numerous examples to suggest that they practiced self-regulation, where they established a set routine for their physical activity, avoided excessive sedentary time, and thus heightened their chances of participating in physical activity. As one adolescent stated: “Routine really plays a big role because once you maintain one thing, it stays with you for the rest of your life so yeah.”–Zaid

#### Controllability and competence beliefs

In addition to logistical (e.g., lack of time and practice) and personal (e.g., perceived lack of skills) factors, adolescents shared not having control over their mental health, which was reported to be a major barrier to being active. The following quotes reflect this reality:

“Um, anxiety and, uh, kind of just feeling down. It’s, um, kind of plays a huge role I know, because I’m always nervous, I’m always paranoid. That’s the main thing stopping me from going out. And then there’s the depression, which makes me tired all the time and then it makes me lose my reason to do anything. And, um, yeah it kind of just, um, traps me to doing nothing, just staying inside […] School isn’t really that bad anymore, I guess. It just, everyday, it seems to get worse for some reason. I don’t really know why, it’s just kind of in my brain.”–Dipti

“Just anxiety in general because sometimes it just makes me not want to get out of bed because the world is a mess and that kind of thing.”–Ace

According to adolescents, and as can be seen by the following quotes, they further lacked control on how to handle their anxiety and depression due to dismissal from parents:

“Um, I have a really low self esteem so I don’t really, um, love myself that much and I don’t feel loved by others that much, either. So that kind of ties into depression and anxiety because no one loves me anymore, is just kind of screaming in the back of my mind […] They don’t have the time for me anymore and if I try to tell them [parents] something, they always brush it off like it’s nothing, so.”–Dipti

“I try to bring up the thought of me maybe having anxiety too, really, when ‘It’s nothing, get over it, just stop things for a bit’ and I was like cool! Ha-ha! No.”–Ace

Adolescents also described a perceived lack of control over their sedentary activity in the context of not realizing the amount of time spent being sedentary. This can be seen in the quotes below:

“I lose track of time when I’m playing [video games]. It’s kind of hard for me to stop.”–Dipti

“You start playing [video games], and because you’re playing, you become tired. When you get to bed, you can’t sleep. You get up, you play again to make yourself tired again, but with this, you can be here for like 11 hours, 12 hours. You can stay here for an entire evening.”–Dominic

Motivation was described by some adolescents as an important enabling factor of being physically active. They explained how they were motivated either by instant (e.g., enjoyment, feeling energized, relieving stress and anger) or delayed (e.g., avoiding long-term health consequences, losing weight) gratification. For example: “If it’s fun, like… if it’s not fun, I don’t think that I would be interested. I won’t really try to do it.”–Abdi

#### Social relationships and interactions

Many adolescents described barriers to physical activity experienced in social settings related to mental health problems and interpersonal relationships. For example, adolescents described how they avoided physical activity in public for concerns of feeling watched, judged, and embarrassed if unfamiliar with the sport or equipment. Adolescents shared how these feelings were sometimes driven by learned helplessness; that is, previous negative experiences with Physical Education teachers threatening to lower grades when wearing baggy clothes, and peer judgments. Further, some adolescents indicated that the lack of peer and parental involvement in physical activity discouraged them from being physically active. For example: “A lot of my friends have very high metabolism, so it’s like they don’t have to exercise or anything, so we’ll just sit around and watch Netflix so that doesn’t help and that’s pretty much it.”–Chloe

For numerous adolescents, use of digital technology was seen as an escape from reality and an alternative to social interaction. As one adolescent stated: “Yeah, I really don’t like to think about what is going on in real life sometimes, so I have a better life in video games than this one.”–Dipti

Whereas some adolescents discredited verbal encouragement from family and peers regarding food and nutrition, they explained that they benefited from verbal encouragement and support for physical activity. For example, when adolescents were encouraged to commit to organized programs or plans, especially those that were more challenging and competitive, they explained that they were more likely to be motivated to persist with their activities. As one adolescent shared: “If someone told me, go to the gym every second day, I’d be like, why? Then, if someone told me, come to the gym every second day, get help with a personal trainer, and push your limits, that sounds a lot more fun.”–Daniel

#### Accessibility to and availability of opportunities for lifestyle enhancement

In addition to having to depend on parents for transportation, some adolescents noted that the lack of safety of their neighborhood prevented them from performing activities by the home. The weather, with temperatures ranging across the spectrum, was also considered a barrier to being active. For example: “Even though I really don’t because then I complain about it being too cold out because I don’t really like putting all these winter clothes on and having to take them back off, so it’s like even in the winter if the sun is out, it’s not, it’s just I hate the sun. Like I look at it and I shame it, I won’t look at it whatsoever.”–Nixy

Those who had more facilitated means for physical activity (e.g., equipment at home, school gymnasiums) commented that it was advantageous in helping them be active.

### Sleep

#### Self-regulation for behavior change

Adolescents shared examples to suggest a lack of structure was dependent on their schedules (e.g., flexibility during non-school periods or work commitments) or their parental involvement (e.g., limit-setting). As one adolescent admitted when asked about the reason for her delayed bedtime the night before the interview: “I just stayed up and was like on my phone and reading some books and watching TV and then all of a sudden it was three a.m. and I was like oh, I guess I should go to sleep.”–Courtney

Many adolescents described being aware of the influence of screen time on sleep with advice from their HCPs. These adolescents described following a regular sleep schedule by outlining a specific sleep time and digital curfew. Other suggested contributors to better sleep included coping mechanisms via medications or audio books or next-day commitments. For example: “People think I’m completely irresponsible, but it’s summer right now. My parents can’t really make that excuse that I have to wake up early and I have really nothing to worry about, but then when it’s school, I really have a lot of things to worry about, so I try to go to sleep. It’s just one less thing.”- Dipti

#### Controllability and competence beliefs

Adolescents described not having control over their sleep time (e.g., poor bladder control during sleep, anxiety, depressive thoughts, emotional distress). As one adolescent shared: “My sleeping is okay, although sometimes I got like, I wake up and I stay up a few hours, but I think that’s kind of normal […] I stayed up a bit later. That’s not really a problem because it’s kind of what everyone does.”–Dipti

#### Social relationships and interactions

Adolescents also reported social barriers to sleep, including the use of technology before bed to connect with their social circle or not having any parental restrictions, leading to poor sleep quality and duration. This is demonstrated by the following quote: “Because I don’t, like my parents don’t force me to go to bed so I can just do what I want. I mean they don’t force me to go to bed now because I just tell them that I stuff to do.”–Nixy

This reality is compared with other households, where parents were said to have a more supportive role in reminding adolescents of their commitments and the need for sleep to be energized.

## Discussion

In our multi-centre, qualitative study, adolescents receiving multidisciplinary clinical care for pediatric weight management identified numerous barriers to and enablers of adopting lifestyle behavior changes related to nutrition, physical and sedentary activities, and sleep. These spanned the following themes: physiological mechanisms and physical health status, self-regulation for behavior change, controllability and competence beliefs, social relationships and interactions, and accessibility to and availability of opportunities for lifestyle enhancement. Among the range of barriers and enablers that adolescents reported, we identified three common factors that positively or negatively influenced adolescents’ ability to adopt lifestyle behavior changes, including the degree of controllability, impact of mental health, and social pressures related to weight management. Our findings show that these issues that may not be traditionally considered as primary for adolescents’ efforts in weight management were highly prevalent. The experiences shared by adolescents provide concrete examples of considerations for successful weight management.

Our findings are in line with previous reports highlighting that nutrition and physical activity changes among adolescents with obesity occur within or beyond their perceived and actual control [21]; importantly, our findings enrich our existing body of English and French literature on lifestyle, including information on sedentary behavior and sleep. For example, many adolescents expressed intentions or efforts to adopt healthy lifestyle behavior changes and recognized that their active involvement and motivation were necessary for success in behavior change. However, adolescents reported that their behaviors were equally influenced by external factors, including support and active participation by their social networks, ready access to inexpensive, energy dense foods, and technologies designed for entertaining and minimizing energy expenditure. These results may be supported by numerous behavior change theories, particularly the Social Cognitive Theory [34]. This theory aids the conceptual understanding of behavior change via a triadic reciprocal causation in which behavior (e.g., skills, self-efficacy), cognitive and other personal factors (e.g., knowledge, expectations), and environmental events (e.g., social norms, accessibility) operate interactively [34]; these interactive effects may explain why adolescents’ individual efforts to adopt lifestyle behavior changes had limits, particularly within a sociocultural environment that can hinder the adoption of healthy lifestyle habits.

Mental health is a priority area for individuals living with obesity [35], a finding that was expressed frequently by adolescents in our study and that intertwined with the lifestyle areas that we explored. Specifically, several adolescents described how they felt emotionally distressed from time to time and the negative impact that these emotions had on managing their weight. For example, many avoided physical activity and/or used digital technology to escape their real lives; others had disordered or unhealthy eating habits, which represented ‘avoidance coping’ [36]. Some adolescents also reported that these behaviors, in turn, exerted a negative influence on their sleep, placing them in a vicious cycle that made it challenging to manage their weight successfully. Chronic and poorly managed mental health conditions including anxiety and depression can negatively affect successful weight management [37–38]; therefore, addressing the psychological, emotional, and social well-being in obesity should precede or complement behavior change efforts. HCPs have an important role to play in addressing mental health issues in adolescents. In the treatment of pediatric obesity, expert recommendations [39] point towards the use of cognitive behavioral therapy, a psychological intervention technique with an emphasis on changing unhealthy attitudes, behaviors, and emotions [14]. Beyond tailoring the treatment approach to the individual patient, adolescents’ social networks, including family members, peers, and other important individuals in their life, are important components of any intervention strategy designed to address healthy lifestyle habits [40]. To date, most interventions designed for managing obesity in adolescents have included a measure of obesity as the primary outcome. When included, psychosocial health is often ranked as a secondary outcome. Given adolescents’ reports in our study, the difficulty in achieving successful weight loss over the long-term, and the increasing academic, clinical, and real-world recognition of broader concepts regarding health and well-being, lifestyle-based interventions for behavior change should evolve to emphasize outcomes beyond weight status to include mental health as a primary intervention focus and outcome [41].

While some adolescents in our study had a positive social network, others lacked support and felt judged negatively or pressured by family members and peers. Adolescents reported how others (especially family members) made unsolicited direct or indirect comments on their behaviors, which led to feelings of shame and exacerbated unhealthy lifestyle choices they made (e.g., pressures to eat unhealthy foods, avoid physical activity, and delay bed times to be present on social media). A number of studies have demonstrated consistently that weight-based stigmatization, perceived or real, can worsen unhealthy eating habits [42], cause negative attitudes about sports [43], and lower participation in physical activity [44]. Weight-based stigmatization affects the emotional well-being of individuals living with obesity [45] and contributes to poor body image and impaired psychosocial functioning [46]. This may help to explain why many adolescents in our study used digital media as their primary means of interacting with others. Treatment options that address adolescents’ use of and interest in technology may be of value in this context. Indeed, reduced anxiety, depression, and stress have all been observed among adolescents participating in interventions delivered using technology [47]. Of note, in delivering treatment options using technology, HCPs, researchers, and parents alike can benefit by gaining a better understanding of the risks and potential harms of social media, including privacy issues [48].

Our study has some limitations to acknowledge. For example, most participating adolescents were of Caucasian origin; however, transferability of our findings was strengthened by including both Anglophone and Francophone participants from two geographically diverse provinces in Canada. Further, as a cross-language study that included data collection in both English and French, the language transformation process may raise some methodological concerns. To optimize rigor, we adhered to recommendations [30–34] to ensure that no meaning was lost and provided a detailed description of the translation process that we applied. Given the qualitative nature of our study, adolescents may also have been limited in their ability to remember (recall bias) or articulate some of their experiences because of limited diction or cognitive development. To minimize this possibility, the interviewer provided adolescents with numerous probes and opportunities to refine or expand on their answers at multiple time points during the interview. Finally, while we strived to adopt an inductive approach to data analysis, we acknowledge that data are not coded in an epistemological vacuum and that researchers cannot completely free themselves of their theoretical and epistemological commitments; the researcher who conducted the interviews made note of these matters in her reflexive accounts.

## Conclusions

Our results describe a range of barriers and enablers that may affect adolescents’ ability to adopt healthy lifestyle behavior changes. Through our research, we hope to promote specific avenues for the development and delivery of interventions, such as tailored treatment and inclusion of a focus on psychosocial well-being which may be irrefutable in managing obesity in adolescents. However, governmental intervention in addressing barriers or capitalizing on enablers in the lifestyle areas explored is still needed for any important changes to occur.

## Supporting information

S1 Appendix Patient engagement panel contract. (PDF)
