# Correction: Barriers and enablers for adopting lifestyle behavior changes in adolescents with obesity: A multi-centre, qualitative study

**DOI:** 10.1371/journal.pone.0221141

**Published:** 2019-08-08

**Authors:** Maryam Kebbe, Arnaldo Perez, Annick Buchholz, Tara-Leigh F. McHugh, Shannon D. Scott, Caroline Richard, Charmaine Mohipp, Michele P. Dyson, Geoff D. C. Ball

The fifth author’s name is spelled incorrectly. The correct name is: Shannon D. Scott. The correct citation is: Kebbe M, Perez A, Buchholz A, McHugh T-LF, Scott SD, Richard C, et al. (2018) Barriers and enablers for adopting lifestyle behavior changes in adolescents with obesity: A multi-centre, qualitative study. PLoS ONE 13(12): e0209219. https://doi.org/10.1371/journal.pone.0209219
