# Supplementary material for: Barriers and enablers for adopting lifestyle behavior changes in adolescents with obesity: A multi-centre, qualitative study
Source: PLoS One. 2018 Dec 18;13(12):e0209219. doi: 10.1371/journal.pone.0209219 (PMC6298663; doi:10.1371/journal.pone.0209219)
Supplement: S1 Appendix — (PDF) [file pone.0209219.s001.pdf]

TEENSAID's Patient Engagement Panel *for Teens in Pediatric Weight Management*

TEENSAID Project

TEENSAID is a research project meant to help us understand teens' views related to lifestyle habits and health care. With this information, we can work to improve health services we provide teens and their families.

TEENSAID Purpose

You are the experts of your own lives, experiences, and priorities. We want and need to learn from you to improve the care we provide all teens and families. As a member of our patient engagement panel (PEP), we will ask you to share your thoughts about clinical and research issues related to weight management.

You can expect to:

- Share your experiences in weight management
- Develop research questions that you think are important and interesting to you
- Share suggestions on how studies should be conducted

Why join our PEP?

By joining our PEP, you will:

- Gain experience in planning and conducting health research
- Help plan research projects that meet your interests and needs
- Work with researchers from the University of Alberta to help other teens like you

Ground Rules

As part of our PEP, we expect you to:

- Attend and participate in scheduled meetings (in-person or telephone)
- Show respect for all other members
- Share your views, even if they differ from other members
- Be open-minded to others' experiences, views, and priorities

---

We really value your participation.
To show our appreciation, you will receive
a $25 gift card.

TEENSAID
Sleep, Activity, Inactivity, Diet
